# Classic and Novel Sex Hormone Binding Globulin Effects on the Cardiovascular System in Men

**DOI:** 10.1155/2021/5527973

**Published:** 2021-07-21

**Authors:** Carla Basualto-Alarcón, Paola Llanos, Gerardo García-Rivas, Mayarling Francisca Troncoso, Daniel Lagos, Genaro Barrientos, Manuel Estrada

**Affiliations:** ^1^Departamento de Ciencias de la Salud, Universidad de Aysén, Coyhaique 5951537, Chile; ^2^Departamento de Anatomía y Medicina Legal, Facultad de Medicina, Universidad de Chile, Santiago 8389100, Chile; ^3^Institute for Research in Dental Sciences, Faculty of Dentistry, Universidad de Chile, Santiago, Chile; ^4^Tecnológico de Monterrey, Hospital Zambrano Hellion, TecSalud, Centro de Medicina Funcional, San Pedro Garza García, Nuevo León 66278, Mexico; ^5^Programa de Fisiología y Biofísica, Instituto de Ciencias Biomédicas, Facultad de Medicina, Universidad de Chile, Santiago 8389100, Chile

## Abstract

In men, 70% of circulating testosterone binds with high affinity to plasma sex hormone binding globulin (SHBG), which determines its bioavailability in their target cells. In recent years, a growing body of evidence has shown that circulating SHBG not only is a passive carrier for steroid hormones but also actively regulates testosterone signaling through putative plasma membrane receptors and by local expression of androgen-binding proteins apparently to reach local elevated testosterone concentrations in specific androgen target tissues. Circulating SHBG levels are influenced by metabolic and hormonal factors, and they are reduced in obesity and insulin resistance, suggesting that SHBG may have a broader clinical utility in assessing the risk for cardiovascular diseases. Importantly, plasma SHBG levels are strongly correlated with testosterone concentrations, and in men, low testosterone levels are associated with an adverse cardiometabolic profile. Although obesity and insulin resistance are associated with an increased incidence of cardiovascular disease, whether they lead to abnormal expression of circulating SHBG or its interaction with androgen signaling remains to be elucidated. SHBG is produced mainly in the liver, but it can also be expressed in several tissues including the brain, fat tissue, and myocardium. Expression of SHBG is controlled by peroxisome proliferator-activated receptor *γ* (PPAR*γ*) and AMP-activated protein kinase (AMPK). AMPK/PPAR interaction is critical to regulate hepatocyte nuclear factor-4 (HNF4), a prerequisite for SHBG upregulation. In cardiomyocytes, testosterone activates AMPK and PPARs. Therefore, the description of local expression of cardiac SHBG and its circulating levels may shed new light to explain physiological and adverse cardiometabolic roles of androgens in different tissues. According to emerging clinical evidence, here, we will discuss the potential mechanisms with cardioprotective effects and SHBG levels to be used as an early metabolic and cardiovascular biomarker in men.

## 1. Introduction

The incidence of cardiovascular mortality is higher in men than in women [1–3], and gender differences are highly related to circulating plasma levels of sex-steroid hormones [4, 5]. Estrogens have cardioprotective effects in women prior to menopause, but in adult men, the main gender-related steroid hormone is testosterone [6]. A study from the Mayo Clinic (2018) exhaustively reviewed and analyzed the main clinical publications in the last 10 years related to plasma testosterone levels, testosterone administration therapies, and their impact on the cardiovascular system. Evidence indicates that physiological testosterone levels are beneficial for the male cardiovascular system, while testosterone deficiency is associated with an unfavorable metabolic profile and increased cardiovascular risk [7].

Sex hormone binding globulin (SHBG) transports testosterone within the blood stream and regulates its bioavailability and access to extravascular target tissues [8, 9]. In men, plasma testosterone levels fluctuate throughout life and begin to decrease in middle age and continue to decline with age [4]. Low plasma testosterone levels in men have been associated with the concept of “andropause,” which rapidly has become a worldwide epidemic condition related to an adverse cardiometabolic risk profile [4, 10, 11]. There is substantial clinical evidence indicating that androgen signaling plays a key role for the cardioprotective benefits elicited by physiological testosterone levels in men.

Several cross-sectional and cohort studies have shown that low SHBG levels are associated with an increased risk of developing metabolic diseases [12, 13]. An association has also been described between SHBG actions unrelated to the transport of sex hormones and metabolic disorders. A meta-analysis examining different concentrations of SHBG showed that low levels of SHBG in men are a predictor of metabolic syndrome and type 2 diabetes [14]. Metabolic abnormalities are closely associated with cardiovascular disease [15–17]. Despite previous evidence, a recent cohort study including about 150,000 middle-aged and aged adult men (40–69 years old) concluded that low circulating SHBG levels are associated with diminished mortality in “all-cause” included in this study, particularly those related with cancer and cardiovascular diseases (CVDs). For total and calculated free testosterone, the expected inverse association with SHBG levels was observed only for “all-cause” and cancer mortality and not for CVD deaths [18]. Another cohort study showed, in a group with age ranging from 35 to 80 years, that elevated levels in SHBG were positively associated with increased incidental cardiovascular disease risk in men over 65 years [19]. However, it is still unknown whether changes in the circulating plasma levels of SHBG, the local expression of SHBG and androgen-binding proteins in tissues such as the heart, or the secondary effects of SHBG level fluctuations in free testosterone are responsible for these effects. In this review, we are going to discuss the mechanisms currently proposed for SHBG cardioprotective effects and how the use of circulating SHBG levels can be useful as an early metabolic and cardiovascular biomarker.

## 2. SHBG Is not Only a Passive Carrier for Sex-Steroids

In men, approximately 70% of circulating plasma testosterone binds with high affinity to circulating SHBG, 20–30% to albumin, and the remaining 1-2% circulates in free form [20]. In women, the majority of plasma estradiol and testosterone are bound to SHBG and other proteins and is not bioavailable; only about 2% of these sex hormones are free to bind to receptors and have an impact on the body [21]. Circulating SHBG modulates the level of free sex-steroid hormones that can enter to diverse target cells [22]. The endocrinological concept known as “free hormone hypothesis” states that the “bioavailable” steroid hormone, i.e., the one that has an effect when bound to its receptor, is the unbound or “free” fraction of steroid hormones [23]. However, recent evidence indicates that circulating SHBG not only is a passive carrier of male sex hormones but also actively regulates testosterone uptake and androgen signaling [24]. Because circulating SHBG binds to sex hormones, the relative plasma levels of this protein can modulate the concentrations of sex-related hormones accessible for use by the body, which has an impact on the processes regulated by the sex hormones [25]. SHBG can also release hormones in specific tissues and cells directly, which can influence both production and effects of sex hormones as well as the expression and function of circulating SHBG. Also, sex hormones bound to circulating SHBG can change the affinity of SHBG to its peripheral receptors. Moreover, intracellular expression of SHBG in testicular proximal tubule cells increases uptake of dihydrotestosterone and prolongs the expression of androgen-responsive genes [26].

One case description of a patient with a homozygous missense mutation in SHBG, which abrogates protein secretion in a 27-year-old man showed low total testosterone but normal free testosterone levels. Despite this, no alterations were seen in sexual development. However, fatigue, muscle weakness, and impaired exercise tolerance were part of the patient's symptoms. Faced with normal levels of free testosterone, this phenotype suggests that circulating SHBG may affect tissues in a manner dependent or independent of testosterone [27]. Frairia et al. studied tissue distribution of the SHBG membrane receptor either in estrogen/androgen-dependent tissues and proposed that the actions of SHBG in tissues are not strictly sex-steroid-dependent [28]. There is evidence suggesting that circulating SHBG interacts with specific proteins in the plasma membrane and it can be internalized once it is accumulated [29]. In steroidogenic tissues, SHBG can be internalized to activate transduction signaling pathways different and independent of those induced by the classical action mechanism based on intracellular androgen receptors [30]. There is also evidence indicating that circulating SHBG, through LG domains, binds membrane receptors with tyrosine kinase activity and G-protein-coupled receptors [20]. Functional plasma membrane receptors for SHBG have been identified in cardiac tissue [31], and SHBG is expressed in the myocardium [32].

### 2.1. Circulating SHBG Internalization

Circulating SHBG protein may be internalized through the low-density lipoprotein-related protein 2 receptor also known as megalin receptor in rat yolk cells. Megalin-deficient mice display defects resembling animals treated with androgen receptor antagonists [30]. Megalin is expressed in several tissues including derived cardiac cells [33]. The human megalin promoter gene possesses PPARs-responsive elements, suggesting a metabolic regulation in the protein expression [34]. In fact, megalin expression is reduced in Ren2 rats, a model of metabolic syndrome [35]. Megalin facilitates the uptake of several ligands, many of which are cataloged as intracrine, including SHBG [36]. These extracellular molecules can act by initiating intracellular signals after internalization. The main intracellular target sites described for intracrine actions include the nucleus and mitochondria. In C2C12, a mouse myoblast cell line, megalin KO, decreases the respiratory and glycolytic capacity [37]. Megalin mediates the retrograde trafficking of TGF-*β* and angiotensin II to mitochondria through the retrograde early endosome-to-Golgi transport pathway and Rab32 [37], all of this playing a role in mitochondrial physiology. Whether the metabolic effects of SHBG are related to its retrograde transport and if this trafficking is related to mitochondrial modulation in cardiac cells are unknown.

### 2.2. SHBG as an External Ligand

SHBG activates several signaling pathways depending on a putative membrane receptor coupled to a G-protein [38], increasing the intracellular cAMP levels in COS-1 cells. An increase in cAMP in MCF-7 cells results as a receptor-mediated action of sex-binding protein [39]. Although stimulation of the cAMP pathway has positive effects on cardiac function, long-term activation produces detrimental effects in the myocardium inducing hypertrophy and heart failure [40]. In lymphocytes, incubation with SHBG induces the phosphorylation of ERK and Akt kinases, an effect that is increased by coincubating with estradiol [41]. All these pathways have been implicated as possible targets in metabolic disorders [42–45].

## 3. Signal Transduction Pathways Involved in SHBG Expression

SHBG is a glycoprotein synthesized and secreted by the liver [46–48] that transports sex-steroids (androgens and estrogens) from steroidogenic organs to their target tissues [6, 20, 24, 49, 50]. The structural organization of SHBG genes is evolutionarily conserved and is expressed in most vertebrates [51, 52]. Additionally, SHBG mRNAs exhibit alternative splicing that encodes the androgen-binding protein (ABP) [22, 53], which differs from SHBG mRNAs by the presence of an exon I (exon A) that does not influence the post-translational modifications required for SHBG secretion [54]. In humans and rats, ABP is produced in the liver [22], Sertoli and Leydig cells [55], and cardiomyocytes [31, 56]. The physiological role of ABPs in peripheral tissues remains poorly understood; however, studies indicate that ABPs regulate the local bioavailability of androgens [56–58]. SHBG/ABPs are polypeptides of 43-44 kDa [20, 50]. The steroid binding domain is in the N-terminus, whereas the regulatory domains can interact with a plasma membrane receptor for SHBG [9, 29, 38, 59, 60]. The human SHBG is a polypeptide of 373 amino acids that constitute a tandem repeat of laminin G-like (LG) domains [9].

Locally produced SHBG modulates the expression of androgen-responsive genes in prostate tissue [61]. Expression of SHBG protein may enhance or inhibit the uptake of androgens in a cell- and tissue-specific manner [62]. SHBG exerts protective roles against excessive androgen exposition during embryonic and fetal cardiogenesis [63]. Hepatic secretion of SHBG is controlled by circulating sex-steroid levels [64, 65]. Others have argued that higher levels of circulating SHBG are compensated *in vivo* by hypothalamic-pituitary feedback, resulting in higher total sex-steroid concentrations [66]. This is a controversial point, and there is a continuing debate over whether—and by which mechanisms—circulating SHBG regulates total, free, and/or bioavailable sex-steroid concentrations and their physiological responses. Expression of ABP has been described in the human heart, and it has been suggested that this protein influences the bioavailability of gonadal steroids in the myocardium [31].

Transcriptional regulation of SHBG has been mainly studied in fetal liver and hepatocyte cell lines [67]. SHBG protein expression is controlled by peroxisome proliferator-activated receptor *γ* (PPAR*γ*), a coactivator for several nuclear transcription factors, including the androgen receptor [68]. PPARs regulate cell metabolism and improve ATP generation [69, 70]. A key metabolic regulator is AMP-activated protein kinase (AMPK), which acts as an energy sensor [71]. AMPK/PPPAR interaction is critical to upregulate SHBG expression by controlling hepatocyte nuclear factor-4*α* (HNF4*α*) [72]. HNF4*α* affects the transcription of many genes involved in lipid metabolism, and this fact may contribute to explain the reported correlations between circulating SHBG levels and lipid metabolism, glucose metabolism, and in consequence to cardiovascular risks [73, 74]. The SHBG promoter contains PPAR-response elements (PPAR-RE), which are required for SHBG expression. PPAR*γ* acts as a transcriptional inhibitor of SHBG gene expression in the liver [75]. Some controversial results have been reported since women receiving troglitazone (a pharmacologic PPAR*γ* agonist) showed increased circulating SHBG plasma levels [76], while treatment of HepG2 cells with rosiglitazone reduced the secretion of SHBG [75]. It has been reported that GW9662, a different PPAR*γ* antagonist, increased the synthesis of SHBG in HepG2 liver cells [74]. A potential explanation was that footprinted region 3 (FP3) in the SHBG promoter gene contain binding domains for HNF4*α*, PPAR*γ*, and RXR retinoic acid receptors [77, 78]. Thus, during normal physiological state, HNF4*α* may bind with high affinity, whereas PPAR*γ* may act as transcriptional inhibitors during alterations of lipid metabolism [75].

Although these studies may homologate certain *in vivo* conditions, they do not consider that gene expression of SHBG can also be regulated by testosterone [64, 65]. In this context, transfection of NB16 cells, which do not express sex-steroid receptors, with a plasmid expressing the androgen receptor showed that incubation with SHBG-testosterone or different hormone or carrier concentrations induces the expression of a reporter androgen-responsive gene in a concentration-dependent manner [30]. Therefore, transcriptional regulation of SHBG seems to be regulated by testosterone levels and transactivation mediated by androgen receptors [64, 65].

**Figure 1:**
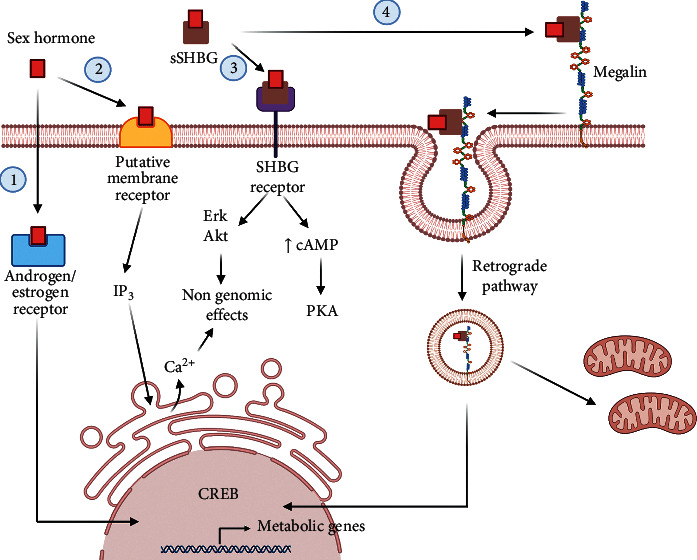
Signaling pathways activated by SHBG. The figure illustrates the action mechanism of SHBG as a hormone carrier and SHBG direct actions. (1) Free circulating androgens and estrogens that correspond to the bioavailable portion of sex hormones can cross the plasma membrane and bind intracellular sex hormone receptors, thus activating the “classic,” genomic sex hormone intracellular pathways. (2) As described in the literature, free circulating sex hormones can also bind to putative membrane receptors activating “fast, nongenomic intracellular signaling pathways.” (3) Another putative membrane receptor, for SHBG, can also activate intracellular signaling pathways, leading to fast, nongenomic effects. (4) The megalin receptor, which induce the internalization of SHBG and a retrograde pathway that affects nuclear and mitochondrial function, can also account for some SHBG-induced intracellular effects.

Adiponectin is a protein produced in the white adipose tissue, negatively related with body mass index (BMI), and their plasma levels are decreased in obese patients. In a study performed in HepG2 human cells, adiponectin increases the levels of SHBG through activation of AMPK-HNF4*α* signaling [79]. In obese patients, there is a chronic low-grade inflammatory state with high levels of TNF*α* and IL-1*β*. TNF-*α* levels are negatively related with SHBG expression. In hepatoblastoma human cells, TNF*α* induces reduction of the levels of SHBG through the activation of NF*κ*B and the inhibition of HNF4*α* P1 promoter activity by the p65 subunit [80]. In streptozotocin-induced diabetic rats, the plasma levels of adiponectin were decreased and have an increase in TNF*α* and IL6 levels [81]. Also, they showed that adiponectin receptor 1 was increased in the heart of diabetic rats, but the levels of adiponectin did not change. In line with this, the systemic levels of adiponectin in plasma were not able to induce the signaling by adiponectin receptor 1, showing a decrease of pAMPK and GLUT4 expression in cardiomyocytes [81]. This information can be hypothetically related with SHBG levels in the heart and plasma of obese and diabetic patients because they share the same signaling factors (Figure 1).

In cardiomyocytes, it has been reported that testosterone activates AMPK to modulate energy production through GLUT4-dependent glucose uptake [82]. AMPK interaction is critical for upregulating SHBG expression in HepG2 cells [79]. Furthermore, upstream AMPK regulates the activity of PPAR-alpha [83]. In the nucleus, androgen signaling stimulates PPAR activity and peroxisome proliferator-activated receptor-*γ* coactivator 1*α* (PGC-1*α*) to increase the expression of various nuclear-encoded metabolic genes, including oxidative phosphorylation genes [84, 85]. Since cardiac cells express SHBG and HNF4*α* gene [86], and testosterone activates the transcriptional machinery to express SHBG protein, including PPAR, AMPK, and HNF4*α* [31, 32, 87], we hypothesize with the possibility that these pathways may be activated to induce cardiac SHBG expression. Transcriptional regulation of SHBG expression involving the AMPK/PPARs pathway also modulates metabolic networks for fatty acid and glucose metabolism during adaptive cardiomyocyte responses [88]. In addition, the protein deacetylase sirtuin 3 (SIRT3) plays cardinal roles in modulating the metabolic network of fatty acid and glucose metabolism for ATP production [89, 90]. Notably, SIRT3 favorably modifies cellular mechanisms implicated in cardiovascular diseases [91, 92]. SIRT expression is controlled by PGC-1*α* and AMPK [93, 94] and testosterone activates PGC-1*α* [95], but it remains unknown whether cardioprotective actions of androgens involve SIRT3 signaling.

## 4. Effects of SHBG and Testosterone on Cardiac Function: Mechanistic Evidence from Animal and Human Research

Most of the research linking SHBG and androgens has been focused on their circulating levels. If altered levels of circulating SHBG are causally related to high cardiovascular risk, raises the question, what is the potential mechanism?

In humans, plasma SHBG levels are influenced by nutritional state, metabolism, and hormonal factors [9, 58, 96, 97]. Patients with obesity and insulin resistance show reduced circulating SHBG levels [74, 98]. Importantly, circulating SHBG levels are strongly correlated with plasma testosterone concentrations and low testosterone levels are strongly associated with metabolic disorders [99–101]. In addition, decreases in SHBG levels are linked to high cardiovascular risk factors [102]. Although metabolic disorders increase the incidence of cardiovascular disease, it is unclear whether SHBG expression has an impact on SHBG receptor signaling or androgen actions in cardiac cells.

Morbidity and mortality in patients with metabolic and cardiac diseases remain high [11] mainly because of the lack of effective cardioprotective strategies to handle cardiometabolic disorders. A 5-year-long follow-up study indicated that men >65 years of age with elevated SHBG and lower total testosterone were independently associated with an increase of both CVD risk and mortality [19, 103]. Circulating SHBG stands as an available marker for assessment of cardiovascular health, especially in the female population, whereas in men similar effects are less known. Future implications of cardiovascular risk assessment and the importance of plasma SHBG in cardiovascular pathophysiology might be even broader since cardiomyocytes of patients suffering from dilated cardiomyopathy produce cardiac SHBG and appear to be internalized, possibly representing a mechanism for delivering sex hormones to the heart [32]. Thus, an interesting concept is that locally expressed SHBG controls testosterone levels in the myocardium to activate additional androgen signaling pathways. Therefore, abnormal locally produced SHBG function might help explain the adverse cardiac metabolic effects of androgen deficiency.

An interesting study in patients with dilated cardiomyopathy demonstrated that cardiomyocytes express an androgen-binding protein, similar to SHBG, and the subcellular distribution matched with androgen receptor location [31]. Moreover, studies in megalin knock-out mice demonstrate a crucial role for this receptor during cardiac development [104]. Immunohistochemical and 3D reconstitution assays showed that these animals had severe cardiovascular anomalies in structures such as aortic arch, common arterial trunk, coronary arteries, and ventricular septum, as well as a marked thinning of the ventricular myocardium [104]. SHBG has been associated with megalin-induced internalization of the protein into the cells; megalin-deficient mice showed defects resembling androgen deficiency [20, 30]. Although reduced levels of circulating SHBG decrease total testosterone levels, the effect of low testosterone on SHBG expression is not fully understood [105].

**Figure 2:**
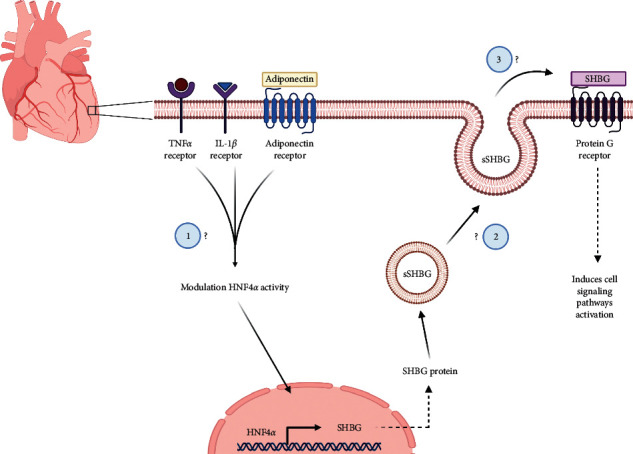
Hypothetical pathways leading to SHBG expression and their modulation in the heart. A hypothetical intracellular pathway for SHBG expression in the cardiomyocytes. The same membrane receptors modulating SHBG expression in the liver could be expressed in the heart. Metabolic cues modulate the activity of the transcription factor hepatocyte nuclear factor 4-*α* (HNF4*α*), which leads to an increase in SHBG gene expression. The following are some of the questions that arise relating to the possible production and secretion of SHBG as an endocrine or paracrine mediator: (1) Can TNF*α*, IL-1*β*, and adiponectin modulate SHBG expression in cardiac tissue? (2) Is the heart involved in the release of soluble SHBG in a paracrine or endocrine way? (3) Can soluble SHBG trigger intracellular signaling pathways in the heart?

Low SHBG levels correlated with measures of heart failure severity and were associated with a higher risk of cardiac death. Interestingly, impaired hepatic SHBG expression impacts testosterone levels, and its deficiency is independently linked to cardiometabolic diseases [106]. In pathological conditions with reduced circulating SHBG levels—such as obesity or insulin resistance—the symptoms of testosterone deficiency in men can be exacerbated [8, 32, 58, 98]. Obesity is considered an independent risk factor for heart failure [17], and mice fed with high-fat diet (HFD) for 16 weeks developed obesity that adversely affects the function and structure of the heart and induces cardiac dysfunction [107]. Moreover, low concentrations of total testosterone and SHBG were strongly associated with an increased likelihood of having metabolic syndrome, independent of other cardiovascular risk factors [108]. It has been proposed that myocytes may produce and secrete ABP in a paracrine manner perhaps to influence the bioavailability of sex-steroids in the myocardium [31]. Low plasma levels of SHBG are associated with several sex-steroid hormone-dependent diseases [109] and have been reported to be an early indicator of cardiovascular risk in individuals suffering from obesity and metabolic syndrome [110–112]. Experimentally, circulating SHBG suppression causes cardiac disorders, partly by mimicking low testosterone level conditions, whereas physiological levels of SHBG and testosterone show cardioprotective effects [14, 25, 73, 102]. Jänne et al. showed that decreased SHBG levels in the kidney by castration can be restored with a treatment with dihydrotestosterone [113]. This experiment shows us that SHBG concentration can be modulated by testosterone but is not fully understood the dependence of testosterone to the variations of SHBG levels and how we can differentiate the effects in cardiometabolic function. Laurent et al. reported that SHBG-tg male mice that overexpress SHBG exhibit an increase in total testosterone concentration compared with wild-type mice, although free levels of testosterone do not change. This result changed when the mice were castrated, eliminating the hypothalamic feedback of luteinizing hormone over Leydig cells, showing a decrease in free testosterone levels [114]. Furthermore, *SHBG-tg* mice showed a significant decrease in the weight of the seminal bladder and levator ani/bulbocavernosus muscle, organs that are sensitive to androgens [114]. In accordance with that, Rastrelli et al. demonstrated that high SHBG levels are related to lower PSA and hematocrit, markers of androgen deficiency, and increase ANDROTEST scores, an androgen-dependent clinical parameter, demonstrating that high SHBG levels in humans can be associated with hypogonadism [115]. In line with this observation, Nokoff et al. reported that boys with obesity have lower levels of SHBG and total testosterone in comparison with normal weight controls, but free testosterone levels do not change [116]. This type of data gives us information about the dependency of testosterone levels with SHBG variations and presents the metabolic and physiological effects that SHBG levels can induce in the body, independent of free concentration of testosterone, challenging the free hormone hypothesis. With these antecedents, the dependence of SHBG on cardiometabolic effects is incomplete and future research is needed to probe this question. An interesting hypothesis is that testosterone levels regulate cardiac SHBG expression to positively influence cardiometabolic responses (Figure 2).

Humanized transgenic mice expressing the human SHBG have been used to study the function of this protein. In this *in vivo* model, SHBG overexpression prevents both the weight increase and fat accumulation induced by high-fat diet. Additionally, SHBG overexpression also abolishes the increase in insulin, leptin, and resistin and protects against high-fat diet-induced obesity [80, 117]. SHBG overexpression does not change food and water intake or intestinal lipid absorption; however, the author did not measure the testosterone levels [117].

On the other hand, elevated levels of circulating SHBG bind more estrogens and may be beneficial by reducing the ability of estrogens to promote breast cancer growth. Also, plasma SHBG levels can directly affect cancer growth [118]. High circulating levels of SHBG have also been associated with better cardiovascular health and metabolic status in postmenopausal women [119, 120]. Therefore, cardiac SHBG expression may be associated with intracardiomyocyte testosterone signaling, allowing cardioprotective effects or, otherwise, producing cardiac dysfunction under metabolic disorder conditions. Thus, expression of cardiac SHBG may restrict the anabolic activity of testosterone on the heart, which impairs cardiac SHBG expression and metabolic adaptations. Then, the relationship between low circulating SHBG and low free testosterone may represent early markers of poor cardiovascular health. Physiological effects of testosterone in cardiac cells includes handling of energy substrates and increased gene expression of key enzymes involved in glucose uptake and glycolysis; and regulation of critical transcription factors related to stimuli that affect cardiomyocyte function.

## 5. Human Diseases and Medications Related to Circulating SHBG

Various human diseases have been associated with altered circulating levels of SHBG, many of which also are linked with high CVD risk (Table 1). In humans, there is diverse information that reflects the bidirectional nature of the relationship between the SHBG levels and metabolic impairment. Different SHBG-gene polymorphisms have been related with metabolic effects in case-control studies. Low levels of SHBG were correlated with cardiac risk since HDL-L levels were lower than the normal threshold in patients with coronary heart disease, and this correlation might be affected by SHBG polymorphism [130]. Carriers of the SHBG polymorphisms rs6257 and rs6259 present a higher risk of diabetes than carriers of other alleles and present low levels of SHBG [131]. Metabolic diseases have been related to abnormal levels of this protein in plasma. SHBG mRNAs in liver and protein levels in serum were lower when the hepatic triglyceride concentration was high and decrease with the increase of body mass index [132].

Metabolic diseases have been related with abnormal levels of this protein in plasma. In obesity, circulating SHBG levels are decreased to 50% in obese adult women (nonmenopause) compared with lean control patients [121, 122]. Also, girls and boys with obesity have near 70% of circulating SHBG compared with nonobese [116]. In patients with diabetes and metabolic syndrome, plasmatic SHBG levels also show decreased levels compared with controls [25, 110, 123]. Laaksonen et al. showed that an entire cohort of patients without diabetes or metabolic syndrome have 34.5 nmol/l of plasma SHBG; nevertheless, some patients that develop diabetes or metabolic syndrome present low levels of SHBG ranging to 26.2 and 28.2 nmol/l, respectively [25]. On the other hand, malnutrition such as anorexia [128] and Kwashiorkor patients with protein and energy malnutrition show increased plasma SHBG levels compared with control normal weight individuals [129]. One example is a longitudinal cohort of patients in whom SHBG levels were evaluated in anorexia and after a treatment to gain weight, showing that the levels of SHBG decrease in the gain weight therapy [129]. This evidence shows that circulating SHBG levels have a negative correlation with the development of obesity/overweight patients and have a positive correlation in malnutrition patients; therefore, plasma SHBG levels are correlating with the nutritional state of patients.

Circulating SHBG is also correlated with endocrinology diseases such as polycystic ovary syndrome (PCOS) and hypothyroidism. In PCOS, the levels of circulating SHBG are decreased compared with control women in a 25%–45% as compared with normal levels [124, 125], whereas in hypothyroidism, the relation is almost the same [126]. Otherwise, in Klinefelter syndrome patients (XXY), there are increased levels of circulating SHBG [127]. In other endocrine diseases such as hypothyroidism, decreased levels of circulating SHBG have been observed, whereas hyperthyroidism leads to increased plasma SHBG levels. This relation has been explained at the level of the transcription factor HNF4*α* being increased by hyperthyroidism [133].

Nokoff et al. showed that obese children in early puberty state have a decrease in the circulating SHBG levels and total testosterone and have an increase in estrone metabolites, probably by the aromatization of androgens in adipose tissue that can lead to develop hypogonadotropic hypogonadism in these boys and affect the reproductive function in the future [116]. There is incomplete information about the impact of plasmatic SHBG and development of cardiometabolic disease in youth, but the evidence showed a correlation between circulating SHBG, hypogonadism, insulin resistance, cardiac metabolism, and dyslipidemia, which are related with low circulating SHBG levels as a result of altered SHBG hepatic production.

Besides hormones and diseases, some medications and dietary compounds alter SHBG liver production [134, 135]. Antiepileptic drugs such as carbamazepine and phenytoin induce an increase in SHBG circulating levels in men and women [134]. Likewise, thiazolidinediones, and oral contraceptives in women, also increase SHBG plasma levels [136]. To the best of our knowledge, only one prospective study has analyzed the effects of changing circulating SHBG levels on cardiometabolic outcomes. In this prospective study, lifestyle interventions directed to obtain favorable changes in circulating levels of SHBG in men and women could not show to influence the risk of developing type 2 diabetes mellitus in the participants [137].

According to our current understanding, the cardioprotective effects of androgens in men have been poorly studied and the deleterious effects exerted by testosterone appear to be controversial. Recent research indicates that administration of testosterone in physiological doses to individuals with metabolic syndrome improves insulin sensitivity and reduces central obesity [138]. Additionally, development of heart failure in individuals with metabolic syndrome is partially reduced by treatment with testosterone in physiological doses [139, 140]. Likewise, subjects with low plasma testosterone levels develop insulin resistance and diabetes, as well as central obesity and heart failure [99, 100, 141, 142]. Moreover, plasma testosterone levels decline with age, while SHBG levels increase, which in turn leads to progression of testosterone deficiency and age-related cardiovascular pathologies [25, 143].

## 6. Conclusion and Future Research

Given the important roles of androgens in normal men physiology, abnormal levels must be considered one of the main causes implicated in several disorders and pathological conditions [108, 144–146]. According to a 2017 update demography report from the American Heart Association, almost one in three adult men have some type of cardiovascular disease [147]. In the context of human disease relevance, the international expert consensus panel that convened in 2015 concluded that there is a need for a major research initiative to explore the possible cardioprotective benefits of testosterone therapy, implying that there is sufficient evidence regarding the safety of testosterone therapy in hypogonadal men and that the direction of future research should be set toward defining suitable therapeutic options for cardiovascular disease [148, 149]. Research in the field of androgen signaling will provide a considerable understanding of the physiological and pathological roles of SHBG and sex-steroid hormones. Thus, an appropriate description of testosterone signaling considering circulating and cardiac SHBG expression might help explain both physiological and adverse cardiac metabolic roles of androgens (particularly androgen deficiency). Research directed to elucidate whether plasmatic and cardiac SHBG expression is associated with physiological testosterone levels could represent novel research approaches to study insulin resistance, obesity, diabetes, and heart failure.

## Figures and Tables

**Table 1:** Pathologies and circulating levels of SHBG.

	Author	Condition	Sex	Age (years)	SHBG (nmol/l)	*p* value	Change
Obesity	Kopelman et al. [121]	Lean control	Women	28	60 ± 8		
		Obese	Women	29	30 ± 4	Non reported	Decreased
	Cupisti et al. [122]	BMI < 25 kg/m^2^	Women	26.41 ± 6.09	53.42 ± 23.1		
		BMI > 25 kg/m^2^	Women	29.23 ± 7.08	30.03 ± 14.52	<0.0001	Decreased
	Nokoff et al. [116]	Normal weight	Women	10	59.5		
		Obese	Women	10	18.5	<0.0001	Decreased
		Normal weight	Men	12	57		
		Obese	Men	12	18	<0.0001	Decreased

Diabetes	Lindstedt et al. [123]	Control	Women	38–60	88 ± 55		
		Diabetes	Women	38–60	55 ± 31	<0.001	Decreased
	Laaksonen et al. [25]	Control	Men	51.3 ± 6.7	34.5		
		Metabolic syndrome	Men	51.4 ± 6.8	28.2	<0.001	Decreased
		Diabetes	Men	52.2 ± 5.6	26.2	<0.001	Decreased
	Ding et al. [110]	Normal	Women	60.3 ± 6.1	36.9 ± 17.4		
		Type 2 diabetes	Women	60.3 ± 6.1	22.3 ± 13.8	<0.001	Decreased
		Normal	Men	63.7 ± 7.6	27.3 ± 10.7		
		Type 2 diabetes	Men	63.7 ± 7.6	19.6 ± 7.2	<0.001	Decreased

PCOS	Ferk et al. [124]	Control	Women	25.3 ± 3.8	61.0 ± 14.7		
		PCOS	Women	24.4 ± 4.4	44.4 ± 19.1	<0.001	Decreased
	Baldani et al. [125]	Control	Women	31.3 ± 4.8	71.6 ± 21.7		
		PCOS	Women	28.3 ± 5.7	38.4 ± 19.9	<0.001	Decreased

Hypothyroidism	Leger et al. [126]	Euthyroid	Boys and girls	7.1 ± 0.5	77.8 ± 7.9		
		Hypothyroid	Boys and girls	7.1 ± 0.5	48.2 ± 6.5	<0.01	Decreased

Klinefelter syndrome	Plymate et al. [127]	Normal	Men	24–40	6.5 ± 1.2		
		XXY Klinefelter's	Men	20–45	16.4 ± 2	Not reported	Increased
	Estour et al. [128]	Normal weight	Women		25.6 ± 62.9		
		Anorexia	Women	20 (14–35)	90.8 ± 32.6	<0.001	Increased

Malnutrition	Pascal et al. [129]	Control	Boys and girls	16 ± 8 months	0.11 ± 0.03		
		Kwashiorkor patients	Boys and girls	20 ± 8 months	0.18 ± 0.07 *μ*mol/l	<0.0005	Increased

The table shows different diseases and some studies that describe the associated circulating SHBG levels. Gender, mean age (±standard deviation), and *p* value change between different conditions are also presented.

## Data Availability

The data used and/or analyzed during the present study are available from the corresponding author on reasonable request.
